# A Web-Based Alcohol and Other Drug Prevention Program (Strong & Deadly Futures) for Aboriginal and Torres Strait Islander School Students: Protocol for a Cluster Randomized Controlled Trial

**DOI:** 10.2196/34530

**Published:** 2022-01-07

**Authors:** Lexine Stapinski, Kylie Routledge, Mieke Snijder, Michael Doyle, Katrina Champion, Cath Chapman, James Ward, Amanda Baumgart, K S Kylie Lee, Maree Teesson, Nicola Newton

**Affiliations:** 1 The Matilda Centre for Research in Mental Health and Substance Use The University of Sydney Darlington Australia; 2 Institute of Development Studies The University of Sussex Brighton United Kingdom; 3 National Health and Medical Research Council Centre of Research Excellence in Indigenous Health and Alcohol The University of Sydney Camperdown Australia; 4 University of Queensland Poche Centre for Indigenous Health University of Queensland St Lucia Australia; 5 National Drug Research Institute Curtin University Bentley Australia; 6 Burnet Institute Melbourne Australia; 7 Centre for Alcohol Policy Research La Trobe University Melbourne Australia

**Keywords:** Aboriginal and Torres Strait Islander, prevention, alcohol, tobacco, substance use, universal prevention, well-being, harm minimization, Indigenous, web-based

## Abstract

**Background:**

There are no available school-based alcohol and drug prevention programs with evidence of effectiveness among Aboriginal and Torres Strait Islander youth. To address this, we codeveloped the *Strong & Deadly Futures* well-being and alcohol and drug prevention program in partnership with an Indigenous creative design agency and 4 Australian schools.

**Objective:**

This paper presents the protocol to evaluate the effectiveness of *Strong & Deadly Futures* in reducing alcohol and other drug use and improving well-being among Aboriginal and Torres Strait Islander youth.

**Methods:**

The target sample will be 960 year 7 and 8 students from 24 secondary schools in Australia, of which approximately 40% (384/960) will identify as Aboriginal or Torres Strait Islander. The study design is a 2-group, parallel cluster randomized controlled trial with allocation concealment. Recruited schools will be block randomized (ratio 1:1), stratified by geographical remoteness, by an independent statistician. Schools will be randomized to receive *Strong & Deadly Futures*, a web-based alcohol and drug prevention and social and emotional well-being program that delivers curriculum-aligned content over 6 lessons via an illustrated story, or *health education as usual* (control). Control schools will be supported to implement *Strong & Deadly Futures* following trial completion. Surveys will be administered at baseline, 6 weeks, 12 months, and 24 months (primary end point) post baseline. Primary outcomes are alcohol use (adapted from the National Drug Strategy Household Survey), tobacco use (Standard High School Youth Risk Behavior Survey), and psychological distress (Kessler-5 Psychological Distress Scale). Secondary outcomes are alcohol and drug knowledge and intentions, alcohol-related harms, binge drinking, cannabis use, well-being, empowerment, appreciation of cultural diversity, and truancy.

**Results:**

The trial was funded by the National Health and Medical Research Council in January 2019, approved by the Human Research Ethics Committee of the University of Sydney (2020/039, April 2020), the Aboriginal Health and Medical Research Council of New South Wales (1620/19, February 2020), the Western Australian Aboriginal Health Ethics Committee (998, October 2021), and the ethics committees of each participating school, including the New South Wales Department of Education (2020170, June 2020), Catholic Education Western Australia (RP2020/39, November 2020), and the Queensland Department of Education (550/27/2390, August 2021). Projected dates of data collection are 2022-2024, and we expect to publish the results in 2025. A total of 24 schools have been recruited as of submission of the manuscript.

**Conclusions:**

This will be the first cluster randomized controlled trial of a culturally inclusive, school-based alcohol and drug prevention program for Aboriginal and Torres Strait Islander youth; therefore, it has significant potential to address alcohol and other drug harms among Aboriginal and Torres Strait Islander youth.

**Trial Registration:**

Australian New Zealand Clinical Trials Registry ACTRN12620001038987; https://www.anzctr.org.au/Trial/Registration/TrialReview.aspx?id=380038&isReview=true

**International Registered Report Identifier (IRRID):**

PRR1-10.2196/34530

## Introduction

### Background

Despite the ongoing impacts of colonization, disempowerment, and inequity, strong cultural and community connections have persisted as sources of resilience for Aboriginal and Torres Strait Islander young people [1,2]. Nonetheless, intergenerational trauma has contributed to poorer emotional and social well-being, with alcohol and other drug (AOD) use identified as both a risk factor for and a consequence of mental illness among Aboriginal and Torres Strait Islander youth [3]. Over the past decade, encouraging trends have been observed with declines in binge drinking (≥5 standard drinks, each containing 10 g of ethanol) and tobacco use reported among young Aboriginal and Torres Strait Islander adolescents [4]. However, Aboriginal and Torres Strait Islander youth continue to experience disproportionate harm from AOD use and, combined with an average earlier age of use [5,6], are at increased risk of substance use disorders later in life [7-9]. To reduce this inequity, it is critical that alcohol and drug prevention initiatives are designed to be culturally relevant for Aboriginal and Torres Strait Islander adolescents to empower them to reach their full potential.

Schools are an ideal setting to deliver alcohol and drug prevention education [10,11]. School-based programs have demonstrated efficacy in preventing or delaying the uptake of AODs and improving attitudes and knowledge of related harms [12-14]. However, a recent international systematic review identified no well-being and drug prevention programs available for Aboriginal and Torres Strait Islander students that are culturally appropriate and effective [15].

### Objectives

To address this gap, *Strong & Deadly Futures* was codeveloped in partnership with an Indigenous Australian creative design agency (Gilimbaa) and with the leadership of Aboriginal and Torres Strait Islander youth (41/77, 53% Indigenous [16]) and staff at 4 Australian secondary schools. The development was informed by stakeholder consultations (17/42, 40% Aboriginal or Torres Strait Islander) and overseen by an advisory group of experts in Aboriginal health, drug prevention, and research (6/16, 38% Aboriginal or Torres Strait Islander). The resulting program, *Strong & Deadly Futures,* is a culturally inclusive curriculum program that aims to reduce harm from AOD use by enhancing coping skills and preventing substance use initiation over the vulnerable adolescent period. The program features web-based delivery to enhance engagement, reach, and implementation fidelity and incorporates core skill development and harm minimization components from the effective *Climate Schools* drug prevention programs [17-25]. It also includes strategies to promote social and emotional well-being, such as coping with stress and effective decision-making. Drawing on feedback from our Aboriginal and Torres Strait Islander stakeholders, *Strong & Deadly Futures* incorporates cultural components to foster pride and appreciation of Aboriginal and Torres Strait Islander culture. Although Aboriginal and Torres Strait Islander culture is central to the program, it was designed to be culturally inclusive so it can be delivered in classrooms with both Aboriginal and non-Indigenous students, reflecting the most common classroom setting experiences of Aboriginal students in Australia. A pilot study showed that *Strong & Deadly Futures* was acceptable and feasible to implement in culturally diverse classrooms, with postprogram improvements observed in students’ knowledge of alcohol and drug harms and overall well-being (unpublished data).

This paper presents a trial protocol (Australian New Zealand Clinical Trials Registry: ACTRN12620001038987) to evaluate the effectiveness of *Strong & Deadly Futures* in reducing AOD use and enhancing the emotional and social well-being of Aboriginal and Torres Strait Islander young people. This will be the first randomized controlled trial (RCT) of a school-based, culturally relevant alcohol and drug prevention program for Aboriginal and Torres Strait Islander adolescents. It is hypothesized that *Strong & Deadly Futures* will be more effective than an active control group (*health education as usual*) in delaying students’ uptake of alcohol and tobacco and reducing psychological distress over a 24-month period. The secondary aims are to examine the effects of *Strong & Deadly Futures* on students’ alcohol and drug knowledge and intentions, cannabis use, binge drinking and alcohol-related harms, well-being and empowerment, appreciation of cultural diversity, and truancy.

## Methods

### Overview

*Strong & Deadly Futures* will continue to be closely developed with Aboriginal and Torres Strait Islander communities. The first phase of the project involves establishing partnerships with local Aboriginal and Torres Strait Islander organizations to conduct community consultations and seek feedback on the program. This feedback will inform adaptations to the program to align it with local priorities and contexts. The second phase involves trialing the adapted program in a school-based RCT. An Aboriginal Reference Group will provide oversight throughout both stages of the project.

### Aboriginal and Torres Strait Islander Leadership

#### Aboriginal Reference Group

An Aboriginal Reference Group will provide oversight and guidance through the consultation, school-based trial, and dissemination phases of the study. The Reference Group will comprise experts involved in the development of the *Strong & Deadly Futures* program and representatives from the local Aboriginal Community Controlled Health Services (ACCHS) or Aboriginal and Torres Strait Islander stakeholder organizations in participating communities. The Aboriginal Reference Group will meet annually throughout the study duration, with the possibility of out-of-session meetings as required.

#### ACCHS Partnerships

In line with best practices in Aboriginal and Torres Strait Islander health research [26], we will ask the local ACCHS or other relevant Aboriginal or Torres Strait Islander stakeholder organizations to consent to the trial proceeding in their community. The ACCHS will be invited to partner with the research team to conduct consultations in their community, and staff will be invited to participate in consultation sessions. A representative from each ACCHS will be invited to sit in on the Aboriginal Reference Group.

#### Local Aboriginal or Torres Strait Islander Facilitators

An Aboriginal or Torres Strait Islander facilitator will be employed in each community to conduct local consultations and support research implementation in schools. Facilitators may be recruited from the local ACCHS or the participating trial school. The consultation role will involve recruiting local community members and organizing and facilitating the consultations. School-based project support will include supporting teachers with the cultural aspects of the program, as well as assisting with survey administration and measurement of implementation fidelity through lesson observations. Facilitators will attend a series of training workshops with the research team before community consultations and commencement of the trial and will be provided with a handbook outlining the research process, methods, and program content. Facilitators will receive regular supervision and mentoring from the research team and will be provided with opportunities to participate in conferences and workshops throughout the study.

### Phase 1: Community Consultations

Consultations will be conducted in each community to obtain local feedback to inform adaptations to the program to align with the local context.

#### Participants and Recruitment

Within the local community of schools enrolled in the trial, facilitators will conduct 2 separate consultation sessions with (1) Aboriginal or Torres Strait Islander adults and (2) Aboriginal or Torres Strait Islander young people aged 12-16 years. Participants will be recruited using a variety of channels, including through the local ACCHS, word of mouth, social networking, and by distributing flyers at community events and health or youth services. Written consent from adults, young people, and parents or carers of young people will be obtained before the consultations.

The research team will also conduct 1:1 semistructured interviews with 1-2 teaching staff from the participating trial school to discuss the feasibility and acceptability of the program in the classroom. Written consent will be obtained before the interviews.

#### Procedure

Consultations with Aboriginal and Torres Strait Islander community members will be conducted using a culturally appropriate research yarning method [27,28]. Research yarning will involve obtaining stories and experiences related to the social and emotional well-being of young people in the community and feedback to inform tailoring of the program to the local context. The yarning circles will be conducted by the local facilitator and audio-recorded if the participants consent. Where possible, a member of the research team will also attend to take notes. If preferred by the community, consultations will be conducted with participants and facilitators of the same gender. Furthermore, 1 or 2 teaching staff from each school will also participate in 1:1 semistructured interviews, conducted on the web by the research team, audio-recorded (with permission), and transcribed.

#### Consultation Feedback and Program Adaptation

Once the community consultations are complete, the research team will synthesize the feedback and create local adaptations of the program in conjunction with oversight and guidance provided by the Aboriginal Reference Group. Program adaptations may involve changes to illustrations, cultural elements, and language. However, the adaptations will not alter the core prevention, harm minimization, and educational components of *Strong & Deadly Futures*, which are based on a large body of existing evidence regarding effective prevention strategies in mainstream and Indigenous populations. A summary of the consultation feedback and the resulting changes will be disseminated by facilitators to the local community.

### Phase 2: School-Based Cluster RCT

#### Participants and Setting

Participants will be year 7 and 8 students from 24 government, Catholic, and independent schools in New South Wales (NSW), Queensland (QLD), and Western Australia (WA), Australia. Students in year 8 (aged approximately 12-13 years) will be the primary target group for program implementation. However, schools will also have the flexibility to enroll year 7 students owing to diversity in community needs expressed during our formative consultations [16] and variations in school and class sizes, including combined grades in smaller schools.

#### Study Design and Randomization

**Figure 1:**
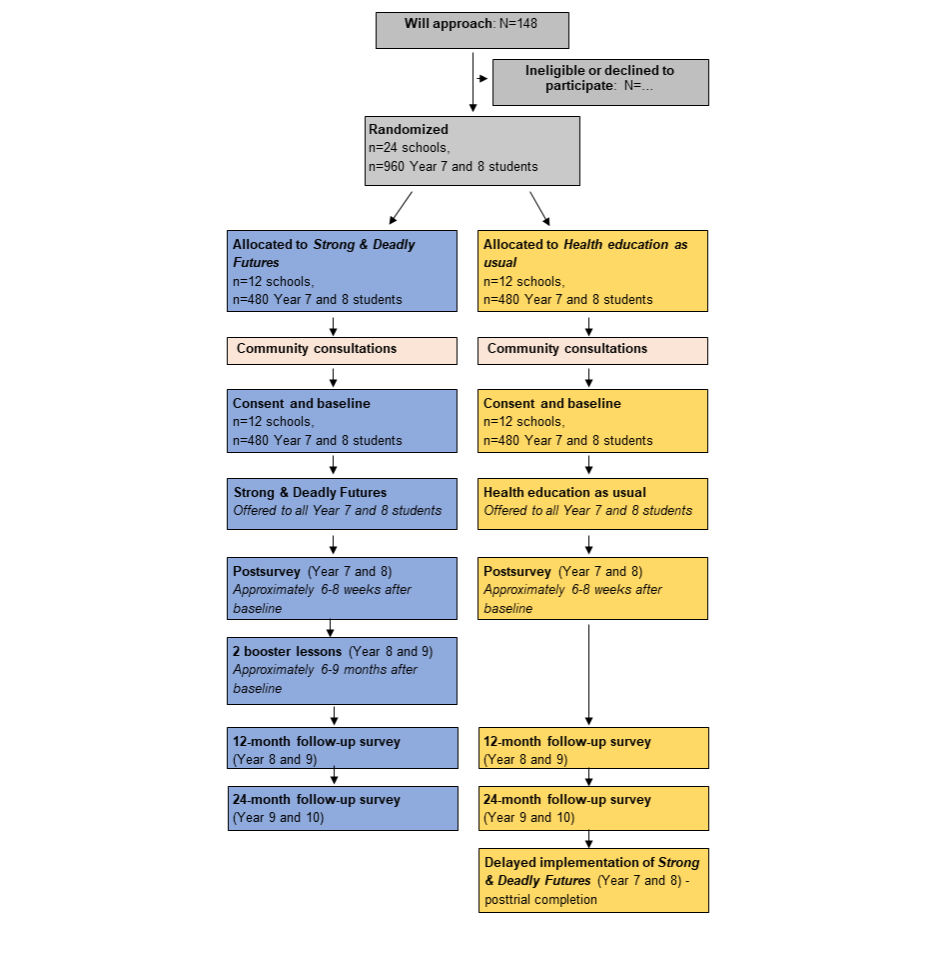
Anticipated *Strong & Deadly Futures* trial flow based on the CONSORT guidelines.

This study will use a 2-group, parallel cluster RCT design. Cluster randomization by schools will be implemented, as randomizing individual students within schools would risk contamination of the control group because of student communication and peer influence effects. Recruited schools will be block randomized (ratio 1:1), stratified by geographical remoteness, to the *Strong & Deadly Futures* group (to implement the program in 2022) or the active control condition (*health education as usual* group). Allocation will be concealed and implemented by an independent statistician using the *blockrand* function in R (R Foundation for Statistical Computing) [29]. Remoteness will be classified using the Remoteness Areas structure within the Australian Statistical Geography Standard [30] and defined as metro or inner regional (major cities and inner regional) and outer regional or remote (outer regional, remote, and very remote). Randomization will occur as soon as a school is recruited and before the community consultations. To ensure that consultations are not influenced by randomization, consultation protocols will prescribe set questions for all sessions. If possible, researchers, facilitators, and participants will remain blind to allocation for the consultations; however, this will depend on consultation timing because of the requirement for schools to have sufficient time to plan class schedules. As schools and teachers play an active role in implementing the program, all schools, facilitators, and researchers will be unblinded before the trial. An overview of the study design is shown in Figure 1.

#### Sample Size Calculations

The sample size has been calculated to account for cluster randomization using the methods developed by Heo and Leon [31] to detect group-by-time interactions in longitudinal cluster RCTs. To adequately power the trial for comparisons among Aboriginal and Torres Strait Islander students, a minimum of 264 Aboriginal or Torres Strait Islander students from 22 schools is required (ie, 12 Aboriginal or Torres Strait Islander students per school). This would achieve 80% power to detect a standardized between-group mean difference of 0.3 (*P*=.05) in primary alcohol (frequency of drinking a standard drink in the previous 6 months), tobacco (frequency of smoking a cigarette in the previous 6 months), and psychological distress (Kessler-5 Psychological Distress Scale) outcomes at the end of the trial with 4 measurement occasions. No effect size information was available for school-based prevention for Aboriginal and Torres Strait Islander students; thus, the estimated effect size of 0.3 was based on effects found for continuous alcohol outcomes in previous school-based prevention trials in mainstream populations [10,32]. Accounting conservatively for a higher student attrition rate than that observed in these trials of 30%, we will recruit 24 schools with an average of 16 Aboriginal or Torres Strait Islander students per school, and a total of 40 students per school. This totals 384 Aboriginal or Torres Strait Islander students, and a total sample of 960 students.

#### Inclusion Criteria

Eligible schools will have at least 12 Aboriginal or Torres Strait Islander students per grade in 2021. Although outcomes will be measured for all students, the primary analyses will be conducted among the Aboriginal subsample in line with the focus of the study. The recruited schools are expected to vary from approximately 10% to 100% of students identifying as Aboriginal or Torres Strait Islander. All students in year 7 and 8 attending the participating schools in 2022 will be eligible to participate, provided they provide opt-in consent and their parents provide opt-in or opt-out consent (dependent upon ethical requirements; see *Informed Consent*).

#### Procedure

##### Recruitment

We will recruit 24 schools across NSW, WA, and QLD during 2021. Researchers will identify and approach eligible schools using data from the MySchool website [33] beginning with those who have previously expressed interest in participating. School principals and health education staff will be emailed an invitation letter detailing the study aims and procedure, followed up by phone calls or emails from the research team.

##### Informed Consent

Once recruited, principals will be asked to provide written consent for the school to participate in the trial. Schools will distribute information and consent forms to parents electronically or by hard copy and will be required to obtain opt-in (QLD public and WA Catholic schools) or opt-out parental consent (NSW and independent schools), depending on the relevant ethical requirements. Where opt-in parental consent is required, school staff members will be reimbursed to follow a standardized script for obtaining verbal consent over the phone in cases where it is not feasible to obtain written consent from parents. Students will be provided with electronic participant information statements and consent forms before survey commencement. Only students with parental consent and who have assented will complete the survey assessments; however, all students in *Strong & Deadly Futures* schools will participate in the program, as it will be delivered as part of their usual drug education curriculum. Year 7 and year 8 health teachers will also be asked to consent to completing web-based logbooks as a measure of implementation fidelity and to permit facilitators to record their observations from the *Strong & Deadly Futures* lessons.

#### Strong & Deadly Futures Group

*Strong & Deadly Futures* is a 6-lesson web-based social and emotional well-being and AOD prevention program that aligns with the Australian year 7 and 8 curriculum and the NSW Stage 4 Syllabus for Health and Physical Education. Each lesson consists of a 10-minute illustrated story, a selection of classroom activities, and teacher and student lesson summaries [16]. Lessons take approximately 45-60 minutes to complete and are optimally delivered once a week for 6 weeks. Program content is based on the established harm minimization [34] and social influence [35,36] approaches to AOD prevention and targets key risk and protective factors identified in our systematic review of effective substance use prevention among Indigenous youth [15]. The individual lesson content and targeted factors are detailed in Multimedia Appendix 1. In brief, the 6 lessons address the following key learning outcomes:

1. Coping with psychological distress, seeking help, and building self-efficacy;
2. AOD education: short- and long-term consequences, harm minimization, helping and coping with other people’s AOD use;
3. Corrective information about normative peer alcohol and tobacco use, finding accurate information about AODs;
4. AOD refusal strategies, coping with peer pressure;
5. Positive alternatives to AOD use, role models.

Students and teachers can access *Strong & Deadly Futures* program content by creating accounts on the program website. Student accounts provide access to the illustrated stories and student lesson summaries. Teacher accounts provide access to the stories, teacher summaries, and class activity options (eg, prespecified worksheets, discussion topics, and activities). Teachers are provided with approximately 6 activity options for each lesson, one of which focuses on cultivating awareness of and pride in Aboriginal and Torres Strait Islander culture. Six months after the 6 lessons, 2 booster sessions will be delivered to help students refresh the content. Booster sessions will be delivered 1 week apart and follow the same structure as the original program.

#### Health Education as Usual Group

Participants in the *health education as usual* group will receive their usual curriculum-based health education classes in 6 weekly 45- to 60-minute lessons over a period of approximately 6 weeks. The Australian and NSW year 7 and 8 Health and Physical Education curriculum mandates that AOD use and social and emotional well-being content be implemented. As such, all *health education as usual* schools will implement curriculum-based drug education during the trial, which will naturally vary in the method of delivery, and serve as an *active control*. Alcohol and drug education in control schools may include tailoring for Aboriginal and Torres Strait Islander students and is expected to vary by school. Details of the content taught will be recorded in logbooks. These schools will be supported to implement the *Strong & Deadly Futures* program with year 7 and 8 students after all trial assessments are complete (from 2024).

#### Assessments

Students will complete self-report surveys at pre- and postprogram implementation and at 12- and 24-month follow-up to assess program effects. Given the preventive focus of the trial, the primary end point is 24 months post baseline to coincide with the peak onset of AOD use during middle adolescence [37]. All primary and most secondary measures were administered to students in our *Strong & Deadly Futures* pilot trial. Surveys will be accessed via electronic links and captured using REDCap (Research Electronic Data Capture; Vanderbilt University) electronic data capture tools hosted at the University of Sydney [38]. REDCap is a secure research data collection platform that links participant data over time while maintaining rigorous security controls to protect students’ privacy. Students will complete surveys in class and be assured by the teacher and facilitator that the data they provide are strictly confidential. If students are absent, teachers or facilitators will arrange an alternate time to complete the survey at school. Follow-up surveys will be sent to students automatically by REDCap using the contact details supplied during the first survey. Where ethical approvals permit it, students will enter into a draw to receive an Aus $ 30 (US $ 21.67) Prezzee gift voucher (1 or 2 per school, dependent on school size) per assessment occasion to maximize participant retention. The timeline schedule for trial participants is shown in Table 1.

**Table 1:** Schedule of program implementation and assessments.

	Preprogram survey	*Strong & Deadly Futures*	Postprogram survey	Booster sessions	12-month follow-up survey	24-month follow-up survey
Period	Term 3 or 4, 2022	Term 3 or 4, 2022	Term 3 or 4, 2022	Term 1 or 2, 2023	Term 3 or 4, 2023	Term 3 or 4, 2024
School grade (age group in years)	7 or 8 (11-13)	7 or 8 (11-13)	7 or 8 (11-13)	8 or 9 (12-14)	8 or 9 (12-14)	9 or 10 (13-15)
*Strong & Deadly Futures* group	✓	✓	✓	✓	✓	✓
*Health education as usual* group	✓		✓		✓	✓

#### Demographics

Demographic variables will be recorded at baseline, including gender (*male*, *female*, *nonbinary* or *gender* *fluid*, *different identity*, *prefer not to say*), age (12-16 years), school year (7-8), and whether students identify as Aboriginal (*Aboriginal*, *Torres Strait Islander*, *Aboriginal and Torres Strait Islander*, *not Aboriginal or Torres Strait Islander*). Socioeconomic status will be assessed using the 4-item Family Affluence Scale [39,40].

#### Primary Outcomes

##### Alcohol Use

Alcohol use will be assessed using measures previously used in *Climate Schools* trials [17,21,41], originally adapted from the National Drug Strategy Household Survey [42]. Students will be asked how often they had a standard drink in the previous 6 months (from 1=*Never* to 6=*Daily or almost daily*). This question will be accompanied by a standard drink chart from the Alcohol and Drug Foundation [43] as a guide.

##### Tobacco Use

Tobacco use will be measured using a single item adapted from the Standard High School Youth Risk Behavior Survey [44] that asks how often students had a cigarette in the previous 6 months.

##### Psychological Distress

Students will complete the 5-item Kessler Psychological Distress Scale, which has been validated for use with Aboriginal populations [45,46]. Students will be asked to indicate how often in the previous 4 weeks they experienced symptoms of psychological distress, such as feeling nervous, using a 5-point Likert-type scale ranging from *None of the time* to *All of the time*.

#### Secondary Outcomes

##### Cannabis Use

Any cannabis use in the previous 6 months will be assessed using measures adapted from the National Drug Strategy Household Survey [42] for use in previous *Climate Schools* trials [17,21,41]. This outcome will not be assessed in all schools because of differing ethics committee requirements.

##### Binge Drinking

Binge drinking will be assessed using a measure adapted from the School Health and Alcohol Harm Reduction Project [12] for previous *Climate Schools* [17,21,41] trials. Students will be asked how often they consumed ≥5 drinks in the previous 6 months (on a 6-point scale from *Never* to *Daily or almost daily*).

##### Alcohol-Related Harms

A 9-item version of the Rutgers Alcohol Problems Index [47] abbreviated for use in the *Climate and Preventure* study [48] will be used to measure students’ experience of alcohol-related harms. Students will be asked to indicate how often they experienced a range of alcohol-related harms in the previous 6 months as a result of their drinking, using a 5-point Likert-type scale ranging from *Never* to *More than 6 times*. Examples of items include *Got into fights, acted bad, or did mean things* and *Caused shame or embarrassment to someone*.

##### Knowledge of Harms and Risk Minimization Strategies Related to Alcohol, Tobacco, and Cannabis

Alcohol (11 items) and cannabis (8 items) knowledge will be assessed using items adapted from the School Health and Alcohol Harm Reduction Project [12], as used in previous *Climate Schools* studies [13,21,22,25]. Tobacco knowledge will be assessed using an 8-item scale adapted from questions used in the *Health4Life* study [49], the School Health and Alcohol Harm Reduction Project [12], and the Life Skills Training questionnaire [50]. For all knowledge measures, students will be asked to respond *true*, *false*, or *don’t know* to statements such as *You can get addicted to cannabis*.

##### Intentions to Use Alcohol, Tobacco, and Cannabis

Intentions to use AODs will be assessed using a single item, which asks how likely students are to try alcohol, tobacco or cannabis in the next 12 months, and measured on a 5-point scale from *Very unlikely* to *Very likely*. This measure has been used in previous *Climate Schools* studies [17,21].

##### Psychological Well-being

The well-being of the students will be measured using the 7-item Personal Well-being Index-School Children scale, which has been validated for use with Aboriginal, Torres Strait Islander, and non-Indigenous young people [51,52]. Students will be asked to indicate their agreement with statements such as *How happy are you with your health?* using an 11-point scale ranging from 1=*Very sad* to 11=*Very happy*.

##### Empowerment

Empowerment will be assessed using the 14-item Emotional Empowerment Scale from the Growth and Empowerment Measure, which has been developed and validated for use with Aboriginal and Torres Strait Islander Australians [53]. Students will be asked to indicate their level of agreement with statements according to *the way you usually feel about yourself most of the time* using a 5-point scale ranging from a negative (score of 1) to a positive (score of 5) statement. Example items include scales ranging from *I feel like I don’t know anything* to *I am knowledgeable about things that are important to me* and from *I feel slack, like I can’t be bothered do things even when I want to* to *I am strong and full of energy to do what is needed*.

##### Appreciation of Cultural Diversity

To assess the attitudes of the students toward cultural diversity, the adapted 8-item Diversity Attitudes scale from the Civic Attitudes and Skills Questionnaire [54] will be used. Responses will be on a 5-point Likert scale, ranging from *Not at all* to *Very much*. Example items include *I have a strong interest in hanging out with people from different backgrounds* and *Cultural diversity within a group makes a group more interesting and effective*.

##### Truancy

Days absent from school without explanation will be obtained from schools, where possible and permitted by ethics committee requirements.

#### Process Outcomes

##### Appropriateness and Relevance

Students will be asked for feedback on *Strong & Deadly Futures* in the postprogram survey using a 13-item measure adapted from previous *Climate Schools* studies [17]. Students will be asked to rate aspects of the program (overall opinion, lessons, cartoons, activities, relevance, and helpfulness) on a scale of 1-5. Students will also be asked whether they think the skills and information will help them deal more effectively with peer pressure, stress, and drugs and alcohol in the future. Responses will be on a 4-point scale from *Yes, I think they will help a great deal* to *No, I don’t think they will help at all*.

##### Implementation Fidelity

Teachers in both groups will complete web-based logbooks recording lesson procedures and activities as a measure of implementation fidelity. Teachers in Strong *& Deadly Futures* schools will be asked how the stories were viewed (collectively or individually), which activities were completed, whether any issues were encountered, and to record any adaptations they made to the lessons. Local facilitators in *Strong & Deadly Futures* schools will record their observations of student engagement, classroom activities implemented, and any issues the students experienced during class lessons. Teachers in control schools will record summaries of AOD education delivered.

#### Analysis

Because of the hierarchical nature of the data, outcome analyses will use multilevel mixed effects regression models (modeled using Stata [StataCorp LLC]) and take into account clustering of data at the school level. Multilevel modeling accounts for the expected correlations between repeated measurements from the same individual and between individuals in the same school, which would otherwise violate the assumptions of independence in traditional regression models. The models will take into account individual differences at baseline, estimating participant-specific starting points and change over time from these baseline levels. The randomly allocated groups (*Strong & Deadly Futures* vs *health education as usual*) will be identified by dummy-coding and entered as an independent variable. Hypothesized program effects on alcohol and tobacco uptake, psychological well-being, and secondary outcomes will be assessed by examining the allocated group-by-time interaction effects. Primary analyses will examine program effects specifically among Aboriginal and Torres Strait Islander students, with a secondary analysis conducted on the full sample. All analyses will be conducted according to the intention-to-treat principle, using all available measurements from the participants and according to their allocated group. Missing data will be accommodated based on all available information using maximum likelihood estimation.

#### Data Safety, Monitoring, and Quality Assurance

The principal investigator (LS) and project manager (KR) will take responsibility for the management and quality control of the study data. Web-based survey data will be collected via REDCap, a secure web-based data management and survey platform that complies with Australian standards in security, ethics, and integrity. Survey data will be stored at the University of Sydney, and all database files will be password-protected with only direct research personnel having access to the databases. Research staff with access to the data will have appropriate training to maintain confidentiality, data integrity, and basic data security measures.

#### Dissemination

After all trial assessments are completed, the research team will travel to the participating schools and communities to report and gain feedback on the interpretation of the aggregated findings of the study. A summary of the trial results will also be provided to all participating schools and communities at the conclusion of the study. This report will aggregate findings across the trial and not identify results for any individual school or student. Schools and facilitators will be able to distribute the results to participating students, staff members, and parents. Schools will be given the option of opting in to be acknowledged in publications for their participation in the trial. The results will be disseminated broadly through peer-reviewed publications in medical, health, and education journals. The findings will also be disseminated at Aboriginal-specific forums and events, at national and international scientific conferences, and through webinars and seminars.

## Results

The trial was funded by the National Health and Medical Research Council in January 2019 and approved by the Human Research Ethics Committee of the University of Sydney (2020/039, April 2020), the Aboriginal Health and Medical Research Council of New South Wales (1620/19, February 2020), the Western Australian Aboriginal Health Ethics Committee (998, October 2021), and the ethics committees of each participating school, including the NSW Department of Education (2020170, June 2020), Catholic Education Western Australia (RP2020/39, November 2020), and QLD Department of Education (550/27/2390, August 2021). The projected dates of data collection are 2022-2024, and we expect to publish the results in 2025. A total of 24 schools have been recruited as of submission of the manuscript.

## Discussion

The aim of this paper was to describe the study protocol to evaluate the first web-based well-being and alcohol and drug prevention program for Australian secondary school students that was developed to be culturally appropriate and empowering for Aboriginal and Torres Strait Islander youth. Program effectiveness will be evaluated through a cluster RCT in 24 schools across 3 Australian states. The trial will be powered to detect medium effects (0.3) within the Aboriginal and Torres Strait Islander sample. It is hypothesized that the program will reduce alcohol, tobacco, and cannabis use and improve well-being relative to health education as usual.

Aboriginal and Torres Strait Islander leadership and input have been prioritized since the inception of *Strong & Deadly Futures*, spanning the program planning, development, and now methodology and implementation of the RCT. This continuing priority is reflected through the involvement of Aboriginal investigators (MD and JW) and the strategic oversight and direction provided by the Aboriginal Reference Group. To ensure *Strong & Deadly Futures* addresses the needs of geographically and culturally diverse communities, we will employ a local Aboriginal facilitator to lead consultations with the Aboriginal and Torres Strait Islander community of each participating school. Community members will identify local alcohol, drug, and well-being priorities for young people and provide feedback on the relevance and appropriateness of cultural content, which will be used to adapt the program to the region. Community participation will ensure that the program is aligned with local values and priorities and meets local needs, which is expected to enhance longer-term sustainability.

Participatory research approaches have been recognized for their potential to empower Indigenous communities and improve health disparities [55], but are often challenging to execute and, more often than not, lack methodological rigor [56]. The participatory approach of this study will combine community participation with rigorous evaluation, with the ultimate aim of developing a flexible, robust program model that is adaptable and generalizable for communities across Australia. This study will also address a critical gap in methodologically rigorous evaluation in Aboriginal and Torres Strait Islander alcohol and drug research [57] and contribute significantly to the field of culturally appropriate, evidence-based prevention. In addition, if effective, *Strong & Deadly Futures* will provide teachers with an evidence-based resource for preventing alcohol and drug use among young people that is accessible, engaging, scalable, and easy to implement.

A limitation of the study protocol is the use of self-report measures to obtain data on alcohol and drug use. However, self-report measures have shown good discriminant [58] and predictive [59] validity for alcohol and drug use, and active measures will be taken to mitigate the risk of over- or underreporting through the use of visual standard drink guides and assurances of anonymity and confidentiality. Another limitation is that blinding to group allocation is not possible because the schools and students are actively involved in the program implementation. However, this is a limitation common to trials of school-based prevention programs. It is also expected that there will be variability in the alcohol and drug education delivered by the control schools. All schools are required to implement alcohol and drug content as part of the curriculum, and this variability will be assessed via logbooks and through implementation fidelity checks. Finally, attrition is a potential source of bias common in longitudinal studies. To ensure sufficient power, the sample size has been calculated using a conservative estimate of attrition, and risks associated with missing data will be mitigated through the use of maximum likelihood estimation in the statistical models. To maximize participant retention, a variety of contact details will be collected to follow up on participants (ie, email addresses, phone numbers, home addresses, and parents’ email addresses and phone numbers).

No culturally appropriate school-based alcohol and drug prevention programs have been rigorously evaluated and demonstrated to be effective for Aboriginal and Torres Strait Islander youth. The *Strong & Deadly Futures* program, which was codeveloped in collaboration with an Indigenous creative design agency and with Aboriginal and Torres Strait Islander and non-Indigenous students, has the potential to address this critical need using a strengths-based approach while promoting Aboriginal and Torres Strait Islander culture within culturally and geographically diverse classrooms.

## Appendix

Multimedia Appendix 1 Overview of learning outcomes and targeted risk and protective factors for *Strong & Deadly Futures* lessons.

## Abbreviations

ACCHS: Aboriginal Community Controlled Health Services
AOD: alcohol and other drug
NSW: New South Wales
QLD: Queensland
RCT: randomized controlled trial
REDCap: Research Electronic Data Capture
WA: Western Australia
