# Supplementary material for: A Web-Based Alcohol and Other Drug Prevention Program (Strong & Deadly Futures) for Aboriginal and Torres Strait Islander School Students: Protocol for a Cluster Randomized Controlled Trial
Source: JMIR Res Protoc. 2022 Jan 7;11(1):e34530. doi: 10.2196/34530 (PMC8783274; doi:10.2196/34530)
Supplement: Multimedia Appendix 1 [file resprot_v11i1e34530_app1.docx]

**Multimedia Appendix 1. *Overview of learning outcomes and targeted risk and protective factors for Strong & Deadly Futures lessons***

| **Lesson** | **Module learning outcomes** | **Targeted modifiable factors (↑ promote; ↓ reduce)** |
| --- | --- | --- |
| 1: New Girl at School | *Target: Psychological distress*   - Contributors to stress - Strategies to cope with feelings of stress - Decision making strategies - Different ways to build self-efficacy | - Psychological distress - Strategies to help cope with psychological distress - build self-efficacy |
| 2: Football Game Gets Out of Control | *Substance: Alcohol*   - Alcohol and the law - Standard drinks and drinking guidelines - Short- and long-term consequences of alcohol use - Drink driving and its risks - Harm minimisation strategies for self and others | - Alcohol consequences knowledge - Positive role models |
| 3: The Day After | *Substance: Alcohol*   - Challenging normative perceptions about alcohol use - Reasons why young people do or do not drink alcohol - Finding accurate information about substances online - Reiterating harm minimisation strategies | - Alcohol consequences knowledge - Harm minimisation knowledge - Peer pressure - Perceived peer substance use |
| 4: The Camping Trip | *Substance: Alcohol and tobacco*   - Health consequences of smoking - Reasons why young people do or do not smoke - Drug and alcohol refusal strategies - Alternative activities to smoking and drinking - The importance of Country and culture for Aboriginal and Torres Strait Islander people | - Tobacco consequences knowledge - Connection to culture - Positive role models - Alternatives to substance use - Peer pressure - Perceived peer substance use |
| 5: Worried About my Sister | *Substance: Cannabis*   - Helping someone going through a challenging time - How to avoid other people’s substance use - Consequences of cannabis use - Cannabis and the law - Dependence on cannabis | - Supportive network - Cannabis knowledge - Connection to culture |
| 6: The ‘Big Day in the Park’ Festival | *Substance: Alcohol, tobacco and cannabis*   - Strategies to cope with feelings of stress - Drug and alcohol refusal skills - Helping someone going through a challenging time - Alternative activities to smoking and drinking alcohol | - Supportive network - Recreational alternatives to substance use - Peer pressure |
